# Exploring the relationship between hadith narrators in Book of Bukhari through SPADE algorithm

**DOI:** 10.1016/j.mex.2022.101850

**Published:** 2022-09-09

**Authors:** Rahmadi Yotenka, Sekti Kartika Dini, Achmad Fauzan, Atina Ahdika

**Affiliations:** Department of Statistics, Universitas Islam Indonesia, Yogyakarta, Indonesia

**Keywords:** Data cleansing, Hadith narrator, Sequential pattern mining, SPADE

## Abstract

As one of the law resources of Muslim society, hadith is very important to learn. Unlike most hadith-related research, which studies more about content, we examine the relationship pattern between hadith narrators. In the study of hadith science, a series of hadith narrators who narrate a hadith is referred to as a sanad. This hadith sanad must be connected to the Prophet as the primary source of a hadith. Therefore, research related to the relationship between narrators is fundamental because it affects the quality and validity of a hadith. This paper analyzes the pattern of hadith narrators using Sequential Pattern Discovery using Equivalence Classes (SPADE). We separate the data of the narrators from the content, whereas, in the hadith books we use, the two are still mixed. This study, therefore, provides detailed information on the steps in the analysis of the patterns of hadith narrators. Some of the highlights of this paper are:

• Algorithm 1 provides the detailed steps in data preprocessing to obtain the “clean data” needed in analyzing the pattern of narrator relationships.
• Algorithm 2 provides a detailed description of analyzing the pattern between hadith narrators using SPADE.

## Graphical abstract

**Figure:**
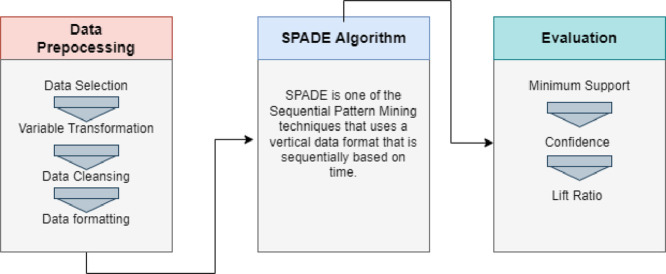

## SPECIFICATIONS TABLE

**Table:** 

Subject Area	*Statistics*

More specific subject area:	*Data Mining*
Method name:	*Sequential Pattern Discovery using Equivalence Classes (SPADE)*
Name and reference of original method:	*Zaki, M.J. (2001). SPADE: An Efficient Algorithm for Mining Frequent Sequences. Machine Learning, 42, 31-60.*
Resource availability:	https://github.com/sutanlab/hadith-api

## Method details

Hadith is one of the main law resources of Muslim society. In the Arabic language, hadith means speech or talk [1]. Thus, in Islamic terminology, hadith consists of words, deeds, and all the circumstances of the Prophet Muhammad, *peace be upon him* (PBUH). Hadith can also refer to Prophet Muhammad's (PBUH) approval or criticism of the actions of other people [1]. Many researches discussed hadith's Matan (redaction) rather than sanad (a series of narrators). This research focuses on the narrators of the hadith, where one of the criteria for the authenticity of the hadith is seen from the profile of the narrators and a continuous sanad. Hadith narrator is the person who narrates hadith consisting of several generations after prophet Muhammad PBUH, i.e., the companions of Prophet Muhammad PBUH, tabi'in, tabi'ut tabi'in, and tabi'ul atba’. The companions of the Prophet Muhammad PBUH, known as as-sahabah, were those who had seen or met him, had held him in high regard while he was still alive, and had also passed away as Muslims. The generation of Muslims known as tabi'in, Arabic for "following" or "successors," followed the companions (as-sahabah) of the Prophet Muhammad PBUH and therefore learned their teachings indirectly. Tabi'ut tabi'in is the generation after tabi'in, while tabi'ul atba’ is the generation after tabi'ut tabi'in. For example, a hadith about the signs of apocalypse is contained in Book of Bukhari number 79, which narrates as follows. “Has told us [Musaddad] said, has told us [Yahya] from [Syu'bah] from [Qotadah] from [Anas ibn Malik], I heard Allah's Messenger PBUH saying: From among the portents of the Hour are (the following): 1. Religious knowledge will decrease (by the death of religious learned men). 2. Religious ignorance will prevail. 3. There will be the prevalence of open illegal sexual intercourse. 4. Women will increase in number, and men will decrease in number so much so that for every man there will be fifty women (ratio wise).” The name of the people in the square brackets is the hadith narrators from many generations after Prophet Muhammad PBUH, which we called sanad.

In maintaining the quality and originality of hadith, hadith scholars have developed stringent criteria and requirements in transmitting hadith. Transmitting hadith is a benchmark and consideration for the quality of the hadith narrated [2]. The classification of hadith based on the quality of the sanad and narrators includes sahih, Hasan, and dhaif hadiths. Sahih hadith is a hadith that can be used as a vital guide for Moslem, with the following criteria. 1) The sanad of the hadith is not broken; in other words, the sanad is continuous from the beginning to the end of the sanad, 2) the narrators are fair –a trait that consistently shows a person who is taqwa and muru'ah (abstaining from inappropriate behavior)–, 3) the narrators can memorize every hadith he heard and can convey at any time. Also, every hadith he narrated is written on the book, which has been checked for truth and is always guarded, 4) the hadith narrated by a reliable narrator does not contradict the hadith narrated by narrators who have a higher level of trust, 5) there is no illat –a subtle defect so that the hadith cannot be accepted–[3,4]. Based on these criteria, it can be seen that the continuity of the sanad affects the quality of a hadith. Several researches have been conducted to classify the hadiths based on their sanad. In its original Arabic, some studies have been conducted to form a full narration tree by parsing the sanad of the narrative text using a shallow parser and context-free grammar (CFG) [5,6]. Another similar study used a finite-state machine to construct the narration tree [7]. Other studies were also conducted using a variety of methods, such as using Principal Component Analysis (PCA) and Backpropagation Neural Network (BPNN) [8], Support Vector Machine (SVM), Naïve Bayes and K-Nearest Neighbor (K-NN) [9], and Genetic Algorithm (GA) [10]. According to the importance of the hadith sanad, a study on the relationship between one narrator and another is important because it relates to the continuity of the sanad in a hadith. A study to find the relationship between narrators has been conducted using the FP-Growth algorithm and ECLAT [11]. Another study has told about making judgments on hadiths according on their sanad (quality) using various computational and Natural Language Processing (NLP) methods [12]. Although previous hadith experts have carried out complex methods based on data and studies of religious knowledge in determining the continuity of the sanad, the development of data analysis science can be applied to study the results of research that hadith experts have carried out.

From a data mining point of view, the relationship among hadith narrators forms an “if-then” rule. The rule, in this case, means that if the narrator X narrates a hadith, then the narrator Y and Z also narrates the hadith in the same sanad. It occurs not only in one hadith but can also be repeated in other hadiths. This pattern is similar to sales transaction data, where customers buy an itemset simultaneously. However, the data of hadith narrators in one sanad has a sequential location according to the generation of the narrators. Thus, to examine more deeply related to the data of hadith narrators, a method to find association patterns that observe the order between items is needed. The conventional association rules cannot accommodate the data pattern which allows transactions that occurred sequentially. One method in data mining to look for patterns of relationships between items that consider the time sequence of events in large amounts of data is the Sequential Pattern Mining [13]. This pattern can be found if large amounts of data and successive events occur several times. The algorithm used in this Sequential Pattern Mining is Sequential Pattern Discovery using Equivalence classes (SPADE). SPADE is one of the Sequential Pattern Mining techniques that uses a vertical data format sequentially based on time [14].

**Fig. 1:**
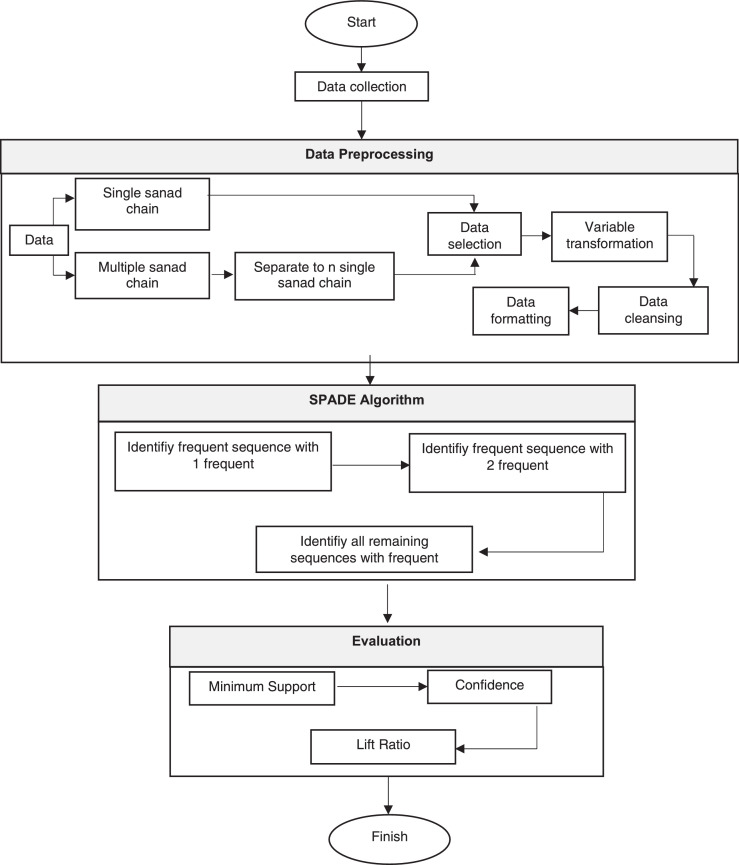
Research flow.

Throughout this paper, we use SPADE to study the relationship among narrators in all hadiths in Book of Bukhari. However, because the hadith data we use is still mixed between sanad and matan, we need to do data preprocessing first. We carry out several stages in the data preprocessing, including selecting the data to be used, transforming the data, cleaning the data chosen from unnecessary elements, and formatting the data to suit the needs in the analysis using SPADE. This data preprocessing stage is reasonably complex because the available information is still very "dirty.” In addition to the data of sanad and matan, which are still mixed, there are also some inconsistent writings of names for the same narrators and writing titles or personal pronouns. This inconsistency can interfere with the pattern analysis that will be carried out because the same narrator can be considered different. Furthermore, there are hadiths which consist of multiple sanad chain which should be separated. Therefore, at this data preprocessing stage, we performed as much detailed data cleaning as possible to maximize pattern analysis results using SPADE. We continue the analysis process using the SPADE algorithm once we get the "clean enough" data. In this analysis stage, we conduct three main processes: determining frequent 1-sequence, determining frequent 2-sequences, and determining the overall frequent k-sequences to obtain patterns of narrator relationships from the books of hadith that we analyzed. After getting the relationship pattern we expect, we evaluate our analysis using the minimum support, confidence and lift ratio. The flow of the research is more simply shown in Fig. 1. Furthermore, we provide the details of each step of our analysis in the following sections, so that our study can be easily reproduced.

### Data collection

Hadith data used in this research are taken from the Book of Bukhari, two of the nine books of hadith, consisting of 6638 hadiths. The raw data can be accessed at https://github.com/sutanlab/hadith-api. We chose the Book of Bukhari because it is one of the two best collections among Sunni Muslims and are hadith collections that have the highest level of authenticity among the nine books of hadith. The raw data still consist of matan and sanad.

### Data preprocessing

**Fig. 2:**
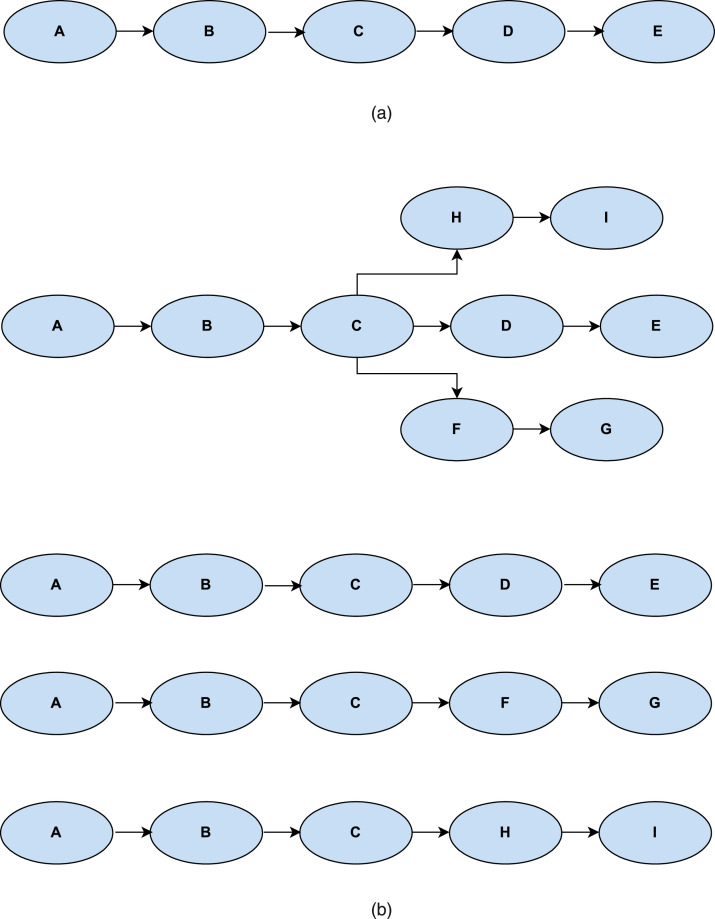
Types of sanad chain; (a) single sanad chain, (b) multiple sanad chain (separated into 3 single sanad chain).

In the database obtained from https://github.com/sutanlab/hadith-api, Book of Bukhari hadith collections are of type *.json*. There are two types of sanad chain, i.e. single and multiple sanad chain. Fig. 2 provides the difference between single and multiple sanad chain.

**Fig. 3:**
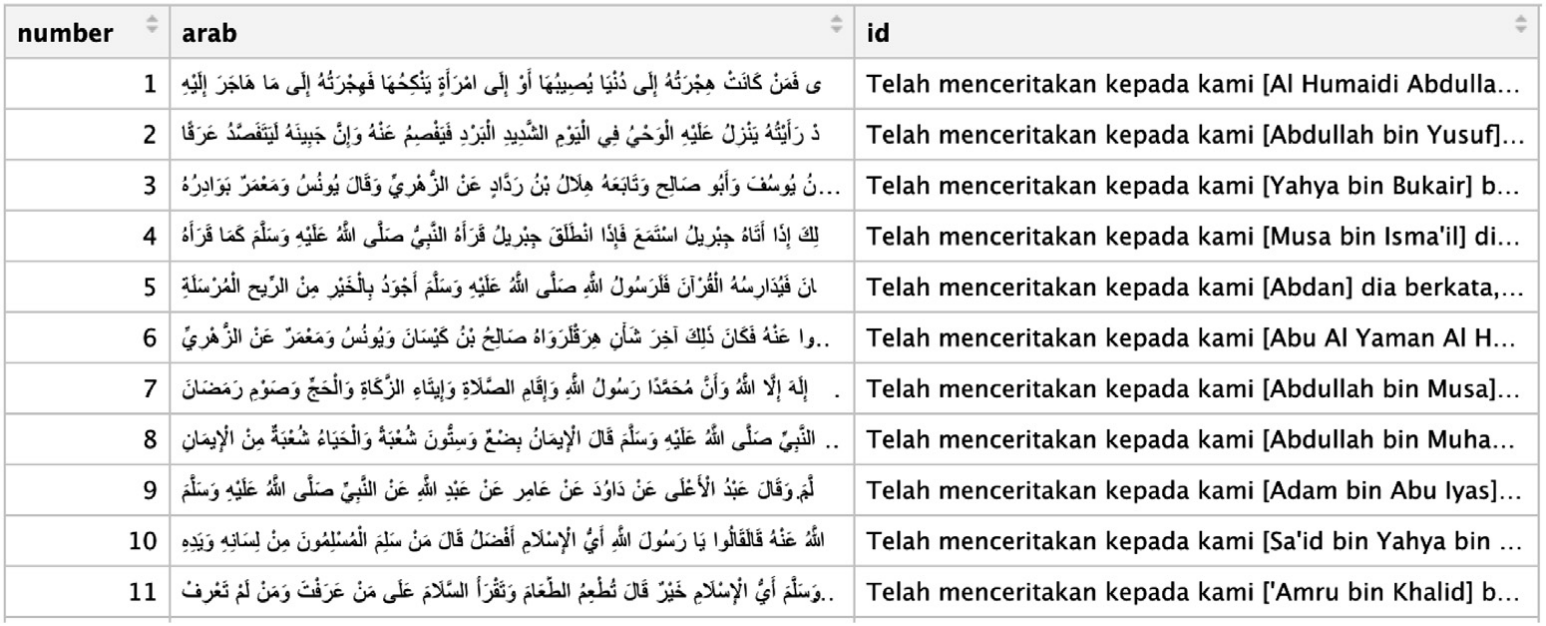
Example of the raw data structure (in Arabic and Bahasa).

For the multiple sanad chain, we separate it into n single sanad chain, where n is the number of chain, and treat them like the other ordinary single sanad chain. After the separation process, we construct the data into a dataframe consist of three variables, i.e., “*number”, “arab”,* and “*id”.* Variable “*number”* is the serial number of hadith from hadith number 1 to hadith number 6638, from the Book of Bukhari. Variable “*arab”* is the matan and sanad, each hadith number in Arabic. Meanwhile, variable “*id”* contains the same thing as variable *arab* but in Bahasa (translation of hadith in Bahasa, an Indonesian language). In this research, we only use variable *number* and *id*. Then, specifically for the variable *id,* which consist of Matan and sanad (sequential flow of hadith narrators), we conduct a data reduction (preprocessing data) into a hadith sanad only.

In general, there are five steps taken in data preprocessing for the hadith data, which are as follows:

1. *Separation process.* The hadith data which consist of multiple sanad chain is separated into n single sanad chain.
2. *Data selection.* The data that will be included in the analysis process is the variable “number” and the variable “id”. Cleaning data for variable “id”. We only used the sanad of hadiths or only the sequences of each hadith number; other data, namely the matan of the hadith or the editorial content of the hadith, will be omitted.
3. *Variable transformation.* We transformed the variable “number” and “id” into a data frame table consisting of i rows and j columns. The number of rows is the number of hadiths in the database. At the same time, the number of columns is the number of names of narrators in each hadith sequentially from the 1st column to the jth column.
4. *Cleaning data for hadith narrators.* In this phase, we performed a cleaning process on the name of the hadith narrators on the variable “id”. We omitted words that are personal pronouns and prayer sentences after the names of the narrators, such as “radliyallahuanhu” (and other similar prayers), “the wife of the Prophet sallallahu alaihi wasallam”, “his uncle”, “my uncle”, “the son of your prophet's uncle”, “nephew”, “his grandfather”, “his father”, “father”, “his mother”, “my mother”, etc. In addition, we removed the spaces in the name of the narrators and changed the name of the narrators in the database to lowercase. All of the cleaning processes in this narrator data are carried out to avoid data redundancies that can affect analytical decisions.
5. *Data formatting.* We modified the data into a transaction data format and formed a vertical sequence table with four columns, i.e., id.[book name], id.rawi.[book name], size.[book name], and rawi.[book name]. The table is created by grouping each hadith narrator labeled by id.[book name]. All narrators in the same hadith are arranged sequentially labeled by id.rawi.[book name], so that a column with a label id.rawi.[book name] is formed as the sequence number of the hadith narrators. A column with label size.[book name] indicates the number of narrators of each row. Table 1 provides examples of the data formatting result.

**Table 1:** Example of data formatting results from Book of Bukhari.

id.bukhari	id.rawi.bukhari	size.bukhari	rawi.bukhari
1	1	1	umarbinalkhaththab
1	2	1	alqamahbinwaqashallaitsi
1	3	1	muhammadbinibrahimattaimi
1	4	1	yahyabinsaidalanshari
1	5	1	sufyan
2	1	1	aisyah
2	2	1	bapak
2	3	1	hisyambinurwah
2	4	1	malik
2	5	1	abdullahbinyusuf
⋮	⋮	⋮	⋮
6638	1	1	abuhurairah
6638	2	1	abuzurah
6638	3	1	umarahbinalqaqa
6638	4	1	muhammadbinfudlail
6638	5	1	ahmadbinisykab

Based on Table 1, the first hadith in the Book of Bukhari (*id.bukhari* 1) is narrated by *umarbinalkhaththab* as a first narrator (*id.rawi.bukhari* 1). Then the second narrator of the first hadith (*id.rawi.bukhari* 2) is *alqamahbinwaqashallaitsi*, and so on. The data formatting step needs to be done as a mandatory step for sequential pattern mining using the SPADE algorithm.

In simple terms, the data preprocessing steps are summarized in Algorithm 1.

**Algorithm 1:** Data preprocessing.

Import data from .json file
fromJSON("[original file]")
Separation process
sep.data <- strsplit(as.character([imported data]$id), split = "#", fixed = TRUE[[1]])
unlist(sep.data, use.names = T)
sep.data
sep.data = as.data.frame(sep.data)
Variable transformation
data.frame(lapply(sep.data, "length<-", max(lengths(sep.data))))
Extract sanad data from the matan
function(i){
gsub("[\\[\\]]", "", regmatches([transformed data][i,3], gregexpr("(?<=\\[).*?(?=\\])", [transformed data][i,3], perl = T))[[1]])
}
Clean hadith narrators data (example for removing personal pronouns)
data.frame(lapply([extracted data], gsub, pattern="radliyallahuanhu", replacement=''))
Data formatting
rotate <- function(x) t(apply(x, 1, rev))
rawi.[book name] <- rotate([cleaned data])
rawi.[book name] <- t(rawi.[book name])
rawi.[book name] <- c(rawi.[book name])
id.[book name] <- rep(1:dim([cleaned data])[1],each=[maximal number of narrators])
id.rawi.[book name] <- c(rep(1:[maximal number of narrators],dim([cleaned data])[1]))
size.[book name] <- c(rep(1,dim([cleaned data])[1]*dim([cleaned data])[2]))
[book name] <- rbind(id.[book name],id.rawi.[book name],size.[book name],rawi.[book name])

**Algorithm 2:** SPADE algorithm.

Import data read.table file
read.table([book name], sep=';')
Data transaction for SPADE Analysis
trans.data <- data.frame(item=rawi$[book name])
trans.rawi <- as(trans.data,"transactions")
transactionInfo(trans.rawi)$sequenceID <- rawi.[book name]
transactionInfo(trans.rawi)$eventID <- id.rawi.[book name]
Determine the frequent 1-sequence (minsupp = 0.001)
cspade(trans.rawi,parameter=list(support=0.001,maxgap=1),control=list(tidLists=TRUE))
Determine the frequent 2-sequence and frequent 3-sequence (minconf = 0.001)
ruleInduction(cseq, confidence = 0.001, control = list(verbose = TRUE))
Determine the strongest rules
sort(subset(rules, subset = lift > 1), by="lift")

### SPADE analysis

After obtaining the “clean data'' of hadith sanad, we conducted a study on the pattern of relationships between narrators using Sequential Pattern Discovery using Equivalent classes (SPADE) –one of the sequential pattern mining algorithms which use vertical data format–. The sequence databases turn into a series of sequences in the vertical data format in the following format; [itemset: (sequence_ID, event_ID)]. In this context, sequence_ID represents the hadith number, while event_ID represents the order of the narrators in the same hadith, which is ordered by generational proximity to the Prophet Muhammad PBUH. In other words, each itemset has a corresponding sequence and event identifier. The event identifier is used as a timestamp of the itemset. A pair of (sequence_ID, event_ID) for each itemset formed an ID_list [14,15]. Table 1 is the example of the vertical data format, where **id.bukhari** column is the sequence_ID and **id.rawi.bukhari** column is the event_ID. Furthermore, **size.bukhari** column represents the number of items for each event_ID and **rawi.bukhari** column represents the item of the hadith narrators.

The steps in finding frequent sequences and determining the frequent rules are [14].

1 Determine the frequent 1-sequence

Frequent 1-sequence from a database with a vertical format (sequence database) can be determined by observing each itemset in the sequence database. Each itemset has an ID_list –a pair of (sequence_ID, event_ID)–. A support value–determined using Eq. (1)–will be added in each new sequence_ID. The sequences with a greater support value than the minimum support will be included in the frequent 1-sequence. For example, suppose that we have the vertical ID_list data transaction provided in Table 2 and we determine the minimum support value of 0.5.

**Table 2:** Example of vertical ID_list data transaction.

Sequence_ID	Event_ID	Size	Item
1	1	1	Narrator_A
1	2	1	Narrator_B
1	3	1	Narrator_C
2	1	1	Narrator_B
2	2	1	Narrator_C
2	3	1	Narrator_D
3	1	1	Narrator_A
3	2	1	Narrator_B
3	3	1	Narrator_C
3	4	1	Narrator_D
3	5	1	Narrator_E

Based on Table 2, we obtain the following support value for each narrator.

• $Support\left(Narrator\_A\right)=\frac{2}{3}$
• $Support\left(Narrator\_B\right)=\frac{3}{3}$
• $Support\left(Narrator\_C\right)=\frac{3}{3}$
• $Support\left(Narrator\_D\right)=\frac{2}{3}$
• $Support\;\left(Narrator\_E\right)=\frac{1}{3}$

Because the predetermined minimum support value is 0.5, therefore, the frequent 1-sequence obtained are <{Narrator_A}>, <{Narrator_B}>, <{Narrator_C}>, and <{Narrator_D}>.

2 Determine the frequent 2-sequences

The data from the frequent 1-sequence are used to search the frequent 2-sequences. Each frequent 1-sequence is combined with other frequent 1-sequence. For example, 1-sequence X is combined with the 1-sequence Y; the possible 2-sequences are X,Y; meaning that X and Y appear together in the transaction. X→Y means that item Y appears after X, and vice versa. For each frequent 1-sequence combination, the ID_list is checked to determine whether the sequence_ID is the same. If the sequence_ID is the same, then it is checked whether the event_ID of 1-sequence X is equal to, less than, or more than the event_ID of 1-sequence Y. If it is the same, then the ID_list is included in 2-sequences X,Y. If event_ID Y is greater than X then the ID_list is included in 2-sequences X→Y, and vice versa. As with frequent 1-sequence, we add a support value to each new sequence_ID. 2-sequences with a support value more significant than the minimum support will be included in the frequent 2-sequences. Based on example in step 1, we obtain the frequent 2-sequences from the combination of Narrator_A, Narrator_B, Narrator_C, and Narrator_D, with the support value as follows.

• $Support\left(Narrator\_A,\;Narrator\_B\right)=\frac{2}{3}$
• $Support\left(Narrator\_A,\;Narrator\_C\right)=\frac{2}{3}$
• $Support\left(Narrator\_A,\;Narrator\_D\right)=\frac{1}{3}$
• $Support\left(Narrator\_B,\;Narrator\_C\right)=\frac{3}{3}$
• $Support\left(Narrator\_B,\;Narrator\_D\right)=\frac{2}{3}$
• $Support\left(Narrator\_C,\;Narrator\_D\right)=\frac{2}{3}$

If we determine the minimum support value of 0.5, then the frequent 2-sequences obtained are <{Narrator_A}, {Narrator_B}>, <{Narrator_A}, {Narrator_C}>, <{Narrator_B}, {Narrator_C}>, <{Narrator_B}, {Narrator_D}>, and <{Narrator_C}, {Narrator_D}>.

3 Determine the frequent k-sequences

The periodic (k−1) sequences with the same prefix is concatenated to find common k-sequences. To discover 3-sequences, for example, combine frequent sequences from 2-sequences with the same prefix, and to find 4-sequences, combine frequent sequences from 3-sequences with the same prefix, and so on. The prefix frequent (k−1) sequences are determined by deleting the final item from the sequence. For example, in 4-sequence A→B→C→D, the prefix is A→B→C. For each of these mergers, there are three possible outcomes, namely:

a. If A,B is combined with A,C, the possible result is A,B,C.
b. If A,B is combined with A→C, then the possible result is A,B→C.
c. If A→B is combined with A→C, then there are three possible outcomes: A→B,C, and A→B→C, and A→C→B.

Furthermore, check the support value for each conceivable outcome. The frequent k-sequence will include k-sequences with a support value more significant than the minimal support. If there are no more frequent (k−1) sequences that may be combined or if frequent sequences are no longer identified, the frequent sequence search is terminated.

The following algorithm summarizes the steps we conducted in analyzing the narrator data using the SPADE algorithm.

## Formation of rules

Sequences that have fulfilled the minimal support limit are used to create rules. There is only one item in a 1-sequence. Therefore, it is not utilized to build a rule. The first item in a 2-sequences is the antecedent, and the second item is the consequent. Meanwhile, in sequences with more than two items (k-sequences), the last item is a consequent, while others are antecedent. For example, in 4-sequences A→B→C→D, the resulting rule is A→B→C⇒D. Next, the confidence value is calculated (Eq. (3)). The accepted rules have a confidence value more significant than the specified minimum confidence. Then for the accepted rule, the lift ratio value is calculated using the formula in Eq. (4).

The level of importance of a rule is determined by the following parameters [16,17]:

1 Support

Support is the percentage of item combinations in the database. Support for the "X⇒Y" rule is the probability of an attribute or set of a co-occurring X and Y attributes. The support value for one item is as follows

(1) $$Support\;\left(X\right)=P\left(X\right)=\frac{n\left(X\right)}{n\left(S\right)}$$

where

P(X): probability of occurrence of X
n(X): the number of transactions containing X
n(S): total transaction amount

The support value of the two items is obtained from the following formula:

(2) $$Support\;\left(X\Rightarrow Y\right)=P\left(X\cap Y\right)=\frac{n\left(X\cap Y\right)}{n\left(S\right)}$$

where

P(X∩Y): probability of X and Y occurred simultaneously
n(X∩Y): the number of the simultaneous occurrences of X and Y

Support in this study is defined as the probability of several items (narrators) narrating the hadith in one sanad (interrelated) from the entire hadith narrated. To produce items from a dataset of narrators that contribute to narrating the hadith, it is necessary to determine the minimum support value. Minimum support is a parameter used as a limit on the frequency of events or support count that must be met by a data group to be used as a rule.

2 Confidence

Confidence is defined as the likelihood of numerous events co-occurring when only one of them is definite. The confidence value of a group of objects is calculated using the formula below:

(3) $$Confidence\;\left(X\Rightarrow Y\right)=P\left(Y|X\right)=\frac{P\left(X\cap Y\right)}{P\left(X\right)}$$

where

P(X|Y): the conditional probability of event Y given by X

The probability of numerous items (narrators) reciting the hadith in one sanad (interrelated) when one of the narrators has undoubtedly reported the hadith is termed as confidence in this study. The minimal confidence value was also calculated in this investigation.

3 Lift Ratio

The Lift Ratio is a metric for determining how potent the rules generated by a sequential pattern mining algorithm are. The lift ratio might be anything between 0 and infinity. Unlike support and confidence, where a minimum value is given, the lift ratio does not have a minimum value. When the lift ratio is less than one, the antecedent rule (X) has a negative impact on the subsequent rule (Y). When the lift ratio is one, the rules frequently appear combined but are separate. An independent rule is when the result (Y) is not reliant on the antecedent. If the lift ratio is more significant than one, the recommended rule is that the antecedent (X) influences the consequent favorably [18,19]. The formula for the lift ratio is as follows:

(4) $$Lift\;Ratio\;=\frac{\;Confidence\;\left(X\Rightarrow Y\right)}{Support\;\left(Y\right)}$$

## Results

With the minimum support of 0.001 and minimum confidence of 0.001, we obtained 1374 rules. We then summarize the rules by selecting those with a confidence value of 1, meaning that all hadiths narrated by Y (consequent), previously narrated by X. Based on this reasoning, we obtained ten rules provided in Table 3.

**Table 3:** Association rules with the minimum support of 0.001 and confidence of 1.

No	Rules	Support	Confidence	Lift Ratio
1	<{Malik bin Al Huwairits}> => <{Abu Qilabah}>	0.0024	1	119.6452
2	<{Abdullah},{Alqamah}> => <{Ibrahim}>	0.0032	1	42.6322
3	<{Abdullah},{Nafi},{Juwairiyah}> => <{Musa bin Ismail}>	0.0022	1	31.4322
4	<{Hammam bin Munabbih}> => <{Ma'mar}>	0.0038	1	29.2047
5	<{Abu Hurairah},{Hammam bin Munabbih}> => <{Ma'mar}>	0.0038	1	29.2047
6	<{Ibnu Umar},{Nafi},{Ubaidullah},{Yahya}> => <{Musaddad}>	0.0027	1	16.4115
7	<{Urwah},{Ibnu Syihab},{Uqail}> => <{Al Laits}>	0.0043	1	16.0563
8	<{Aisyah},{Urwah},{Ibnu Syihab},{Uqail}> => <{Al Laits}>	0.0032	1	16.0563
9	<{Sumayya}> => <{Malik}>	0.003	1	9.8382
10	<{Ibrahim},{Al Hakam}> => <{Syu'bah}>	0.0022	1	9.4617

The first rule is a frequent 2-sequences rule with a support value of 0.0024, meaning that 0.24% or 16 hadiths in the Book of Bukhari are narrated by Malik bin Al Huwairits and Abu Qilabah. The confidence value of 1 means that if Malik Al Huwairits narrated a hadith, then it will also be narrated by Abu Qilabah with a confidence level of 100%. The lift ratio of 119.6452 (greater than one) means that the relationship between the hadith narrators is strong. The historical fact strengthens that the relationship between the two narrators is a teacher-student, where Malik bin Al Huwairits is the teacher of Abu Qilabah. Moreover, Malik bin Al Huwairits is a hadith narrator from the generation of the Prophet's companions.

Meanwhile, Abu Qilabah, whose real name is Abdullah bin Zaid ‘Amru bin Nabil, is a hadith narrator from the generation of tabi'in –early Muslims who lived after the companions of the Prophet and did not experience the life of the Prophet Muhammad–. This fact shows that the relationship between the two hadith narrators is strong. Different patterns are shown by the second, third, fifth, sixth, seventh, eighth, and tenth rules, where the sanad in these rules consist of more than two people. For example, in the fifth rule, Ma'mar narrated a hadith which Hammam bin Munabbih and Abu Hurairah previously narrated. Furthermore, for other rules with more than two narrators, the nature of the relationship is the same as for the fifth rule.

**Fig. 4:**
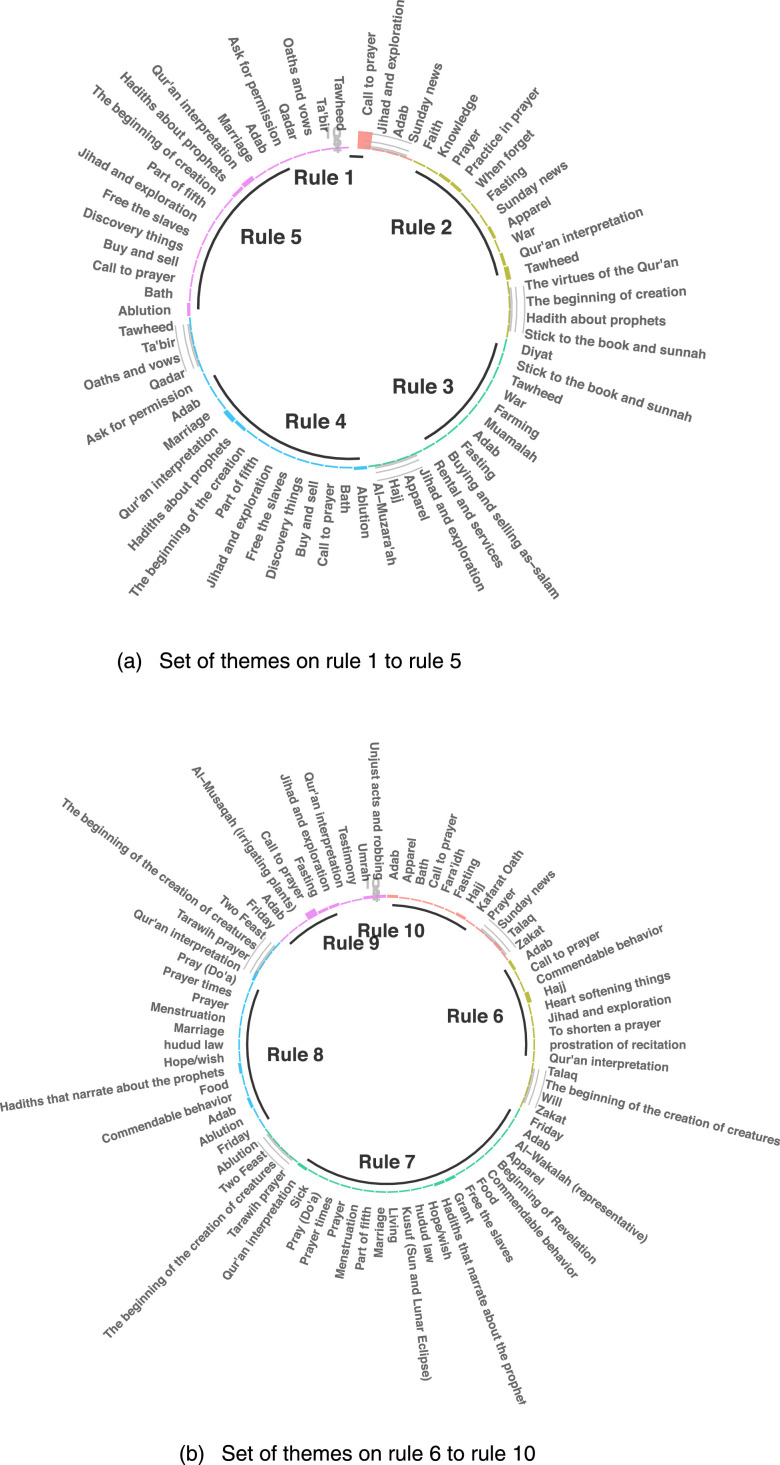
Set of hadith themes narrated by hadith narrators in each rule. (a) Set of themes on rule 1 to rule 5. (b) Set of themes on rule 6 to rule 10.

Based on the acquired rules, we collect the information about the themes narrated by the hadith narrators in each rule and summarize it into Fig. 4.

Fig. 4 shows the distribution of themes in the hadiths narrated by the narrators on the ten acquired rules. Based on the results, we obtain the following information. Rule 1 and rule 9 dominantly narrated the themes of the call to prayer, while rule 2 dominantly narrated about Tawheed. The dominant themes of other rules can be identified from the highest bar in Fig. 4.

## Conclusion and perspective

This research discussed the relationship pattern between hadith narrators in the Book of Bukhari using the SPADE algorithm. This study describes the work steps in detail to easily reproduce this procedure by the related researchers. From 6638 recorded hadiths in the Book of Bukhari, we obtained ten rules which describe the relationship pattern between the narrators, with the minimum support of 0.001 and a confidence value of 1. We also identified the themes and summarized each rule's dominant theme narrated by the hadith narrators.

For further research, the SPADE algorithm can be developed to build web-based application. Users can just enter the name of the narrator along with the name of the book of hadith, also the minimum support and minimum confidence. The output of the web-based application can show the sanad chain of narrators, the rules along with each support value, confidence value, and the lift ratio, they can also obtain what themes were narrated by the narrators. As a convenience for users, the web-based application can also display graphs that illustrate the themes narrated by narrator rules that are formed based on the analysis using SPADE algorithm.

## Declaration of competing interests

The authors declare that they have no known competing financial interests or personal relationships that could have appeared to influence the work reported in this paper.

## References

[1] Mahmoud S., Saif O., Nabil E., Abdeen M., Elnainay M., Torki M. (2022). AR-Sanad 280K: a novel 280k artificial sanads dataset for Hadith narrator disambiguation. Inf.

[2] Kusroni (2016). Mengenal Tuntas Seluk Beluk Periwayatan Hadis. Riwayah J. Stud. Hadis.

[3] A.-M. MA, Ilmu Usul Hadits. Yogyakarta: Pustaka Pelajar, 2005.

[4] Rohman T., Huda U., Hartono (2019). Methodology of hadith research : the study of Hadith criticism metode penelitian hadis : studi tentang Kritik Hadis. J. Hadith Stud..

[5] Azmi A., Bin Badia N. (2010). Proc. 6th Int. Conf. Nat. Lang. Process. Knowl. Eng. NLP-KE 2010.

[6] Azmi A.M., Bin Badia N. (2010). e-Narrator - an application for creating an ontology of Hadiths narration tree semantically and graphically. Arab. J. Sci. Eng..

[7] F. Zaraket and J. Makhlouta, “Hadith narrator chain extraction using arabic morphological analysis,” in *Proceedings of the Twenty-Fifth International Florida Artificial Intelligence Research Society Conference*, 2016, no. December, pp. 256–261.

[8] Nuha U., Rochmawati N. (2019). Klasifikasi Kesahihan Hadits Berdasarkan Perawi Hadits Menggunakan Principal Component Analysis (PCA) dan Backpropagation Neural Network (BPNN). J. Informatics Comput. Sci..

[9] Mohammad Najib S.R., Abd Rahman N., Kamal Ismail N., Alias N., Mohamed Nor Z., Alias M.N. (2017). Comparative study of machine learning approach on Malay translated hadith text classification based on Sanad. MATEC Web Conf.

[10] Najeeb M.M.A. (2020). A novel hadith processing approach based on genetic algorithms. IEEE Access.

[11] Yunantasena D.E. (2020). Analisis perbandingan algoritma fp-growth dan algoritma eclat dalam menemukan pola hubungan antar perawi hadits. UIN Syarif Hidayatullah.

[12] Azmi A.M., Al-Qabbany A.O., Hussain A. (2019). Computational and natural language processing based studies of hadith literature: a survey. Artif. Intell. Rev..

[13] Han J., Pei J., Kamber M. (2011).

[14] Zaki M.J. (2001). SPADE: an efficient algorithm for mining frequent sequences. Mach. Learn..

[15] Ardiansyah R. (2013).

[16] Zhao Y. (2012).

[17] S. Brin, R. Motwani, and C. Silverstein, “Beyond market baskets: general-izing association rules to correlations,” 1997.

[18] Fomby T. (2011).

[19] Zhang C., Zhang S. (2002).

